# Protocol for a Randomized Clinical Trial to Assess the Effects of Extra-Articular Strengthening in Anterior Cruciate Ligament Reconstruction on the Clinical and Functional Condition of High-Performance Athletes

**DOI:** 10.1055/s-0046-1818612

**Published:** 2026-04-22

**Authors:** Thiago Lemos, Conrado T. Laett, José P. G. Aramburu Filho, Geraldo R. Motta Filho, João M. Guimarães, José Leonardo R. Faria

**Affiliations:** 1 Neuromuscular Research and Exercise Physiology Laboratory, Instituto Nacional de Traumatologia e Ortopedia Jamil Haddad (INTO), Rio de Janeiro, RJ, Brazil; 2 Postgraduate Program in Rehabilitation Sciences, Centro Universitário Augusto Motta (UNISUAM), Rio de Janeiro, RJ, Brazil; 3 Hospital Central da Polícia Militar do Estado do Rio de Janeiro, Rio de Janeiro, RJ, Brazil

**Keywords:** functional physical performance, orthopedic surgery, rehabilitation, sports injuries, cirurgia ortopédica, desempenho físico funcional, lesões esportivas, reabilitação

## Abstract

**Objective:**

To describe a randomized clinical trial designed to evaluate the impact of three extra-articular reinforcement techniques combined with anterior cruciate ligament reconstruction (ACLR) on postoperative muscle function, activity levels, and return-to-sport in high-performance athletes. The primary hypothesis is that these techniques will differentially influence joint stability and recovery trajectories.

**Methods:**

A prospective, quasirandomized, single-blind trial intending to enroll 220 athletes, between 18 and 45-years-old, with isolated ACL injuries. Participants will be allocated via block randomization to one of three procedures: lateral extra-articular tenodesis (LET), involving fixation of a iliotibial band strip near the anterolateral ligament (ALL) at 30° knee flexion for rotational stability; single-bundle ALL reconstruction (ALL-S), using a gracilis graft between the femoral epicondyle and Gerdy's tubercle; and double-bundle ALL (ALL-D), employing two graft limbs in a double tibial tunnel located between Gerdy's tubercle and the fibular head, with a single femoral fixation at the anatomical position of the ALL. Assessments will occur preoperatively and at 3, 6, 9, 12, and 24-months postsurgery, including joint laxity, quadriceps morphology, functional performance, and knee isokinetic strength. Furthermore, self-reported outcomes, such as the International Knee Documentation Committee's (IKDC), 36-Item Short Form Health Survey (SF-36), and Tegner scale, will also be collected. Linear mixed-effects models will analyze outcomes, adjusting for meniscal repair status.

**Results:**

Upon completion, this study is expected to identify the most effective extra-articular technique for restoring knee stability and function, potentially guiding surgical decision-making. Our findings may optimize rehabilitation protocols and reduce reinjury rates in athletes.

**Conclusion:**

This protocol describes a comprehensive clinical trial that addresses important gaps in current knowledge regarding optimal surgical approaches for ACLR in athletes.

## Resumo

**Objetivo:**

Descrever um ensaio clínico que pretende avaliar o impacto de técnicas de reforço extra-articular combinadas com a reconstrução do ligamento cruzado anterior (R-LCA) na função muscular, atividade física e retorno ao esporte em atletas de alto rendimento. A principal hipótese é que essas técnicas influenciarão diferentemente a estabilidade articular e a recuperação.

**Métodos:**

Estudo prospectivo, quasi-aleatório e simples-cego pretendendo incluir 220 atletas, entre 18 e 45 anos de idade, com lesões isoladas do LCA. Os participantes serão randomizados em blocos para um dos três procedimentos: tenodese extra-articular lateral (TEL), consistindo na fixação de uma faixa da banda iliotibial próximo à inserção do ligamento anterolateral (LAL) com 30° de flexão do joelho para estabilidade rotatória; reconstrução do LAL com feixe único (LAL-U) utilizando um enxerto de tendão semitendíneo entre o epicôndilo femoral e a tuberosidade de Gerdy; ou LAL com feixe duplo (LAL-D), empregando dois ramos do enxerto com fixações femorais e tibiais separadas. As avaliações ocorrerão no pré-operatório e aos 3, 6, 9, 12 e 24 meses pós-cirúrgico, incluindo lassidão articular, morfologia do quadríceps, desempenho funcional e força isocinética. Além disso, dados autorrelatados, como o da International Knee Documentation Committee (IKDC), 36-Item Short Form Health Survey (SF-36), e a escala de Tegner também serão coletados. Modelos lineares de efeitos mistos serão usados, para análise, ajustando-se ao reparo meniscal.

**Resultados:**

Após a conclusão do estudo, esperamos identificar a técnica de reforço extra-articular mais eficaz para restaurar estabilidade e função do joelho, orientando decisões cirúrgicas. Os achados podem otimizar a reabilitação e reduzir recidivas em atletas.

**Conclusão:**

Este protocolo aborda um ensaio clínico detalhado, referente a lacunas importantes no conhecimento atual sobre as melhores abordagens cirúrgicas para R-LCA em atletas.

## Introduction

Sports injuries often result from acute trauma or chronic overuse, ranging from ligament ruptures and dislocations to fractures and cartilage damage. Knee injuries are the most prevalent, frequently involving anterior cruciate ligament (ACL) tears, meniscal damage, and cartilage lesions. The knee is particularly vulnerable in pivoting sports like soccer and basketball. More specifically, ACL tears often occur when the knee is semi-extended under valgus stress, 1 leading to laxity and proprioceptive impairments. Meniscal damage occurs in 44 to 55% of cases. 2

While conservative management suffices for some injuries, structural damage often requires surgery to restore function and facilitate return-to-sport. 3 Reconstruction is the primary treatment for ACL, sometimes combined with extra-articular reinforcement techniques for greater stability. Since the rise of arthroscopy in the 1990s, techniques have advanced, using grafts from the patellar, hamstring, or quadriceps tendons, 4 Combined ACL reconstruction (ACLR) and meniscal repair yield better outcomes than isolated repair. 5

For high-risk groups (e.g., young athletes, women), extra-articular reinforcement reduces failure rates, as with modified Lemaire tenodesis (LET) and anterolateral ligament (ALL) reconstruction. While LET uses iliotibial band tissue, 6 ALL reconstruction complements ACL surgery. 7 Recent studies highlight the role of anterolateral structures in rotational stability, reducing reinjury rates. 7 However, the efficacy of these methods, with or without meniscal repair, remains under investigation.

This study describes the protocol for a randomized clinical trial designed to evaluate the effects of ACLR with different extra-articular techniques on muscle function and activity levels in high-performance athletes. We hypothesized that these techniques may variably impact joint stability, influencing recovery trajectories. The a priori publication of this protocol ensures transparency, safeguards against reporting bias by predefining primary outcomes, and aligns with best practices in clinical research, as recommended by the Standard Protocol Items: Recommendations for Interventional Trials (SPIRIT) guidelines. 8

## Methods

### Ethical Concerns and Clinical Trial Registry

The study protocol was approved by the local Ethics Committee (CAAE: 84435324.0.0000.5273) on January 29, 2025, and was registered in the national clinical trials registry database (RBR-43j2rh9), with the first version being approved on April 8, 2025. All procedures will adhere to the ethical principles established in the Declaration of Helsinki. Potential participants will receive detailed explanations of the procedures, and written informed consent ( **Supplementary Material 1** ) will be obtained by a research assistant prior to enrollment. Identifiable data (e.g., names) will be replaced with codes and anonymized for publication.

### Trial Design

This prospective, longitudinal, three-arm, quasirandomized, single-blind superiority trial will evaluate high-performance athletes with ACL injuries at an orthopedics referral hospital, following SPIRIT guidelines. 8

**Fig. 1:**
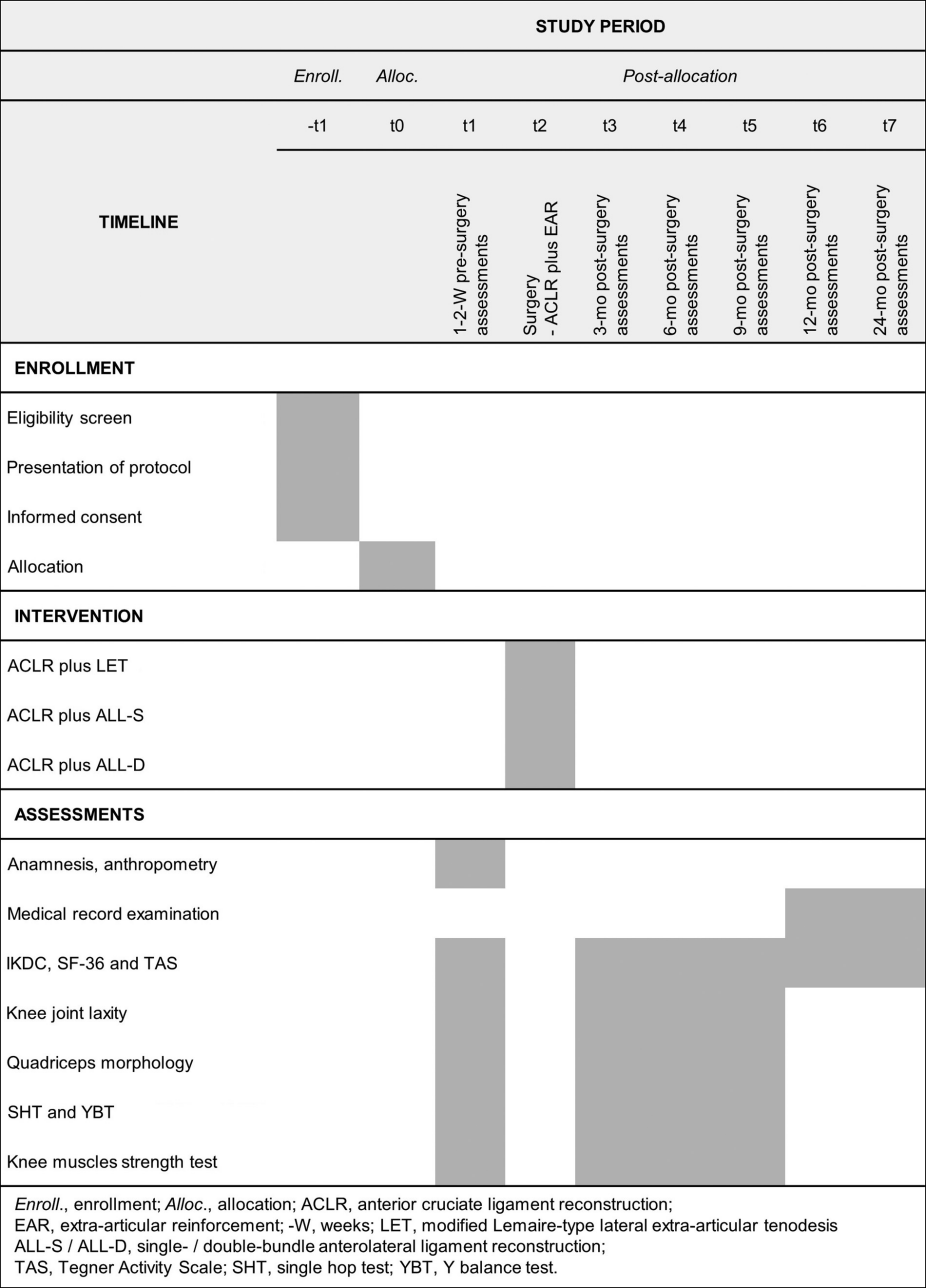
Standard Protocol Items: Recommendations for Interventional Trials (SPIRIT) flowchart of the study procedures. Enrollment, interventions, and assessments are detailed for each study phase.

Participants will undergo on-site assessments at baseline (1–2 weeks before surgery) and at 3, 6, and 9 months after surgery, collecting demographic, clinical, and functional data (self-reported outcomes, knee laxity, quadriceps morphology, strength, and performance tests). Unilateral functional tests will start with the unaffected limb for familiarization, with evaluators confirming participant comfort beforehand. Each visit will last approximately 90 minutes. Telephone follow-ups at 12 and 24 months will track clinical and patient-reported outcomes. Enrollment, randomization, and intervention flow are outlined in Fig. 1 .

Amendments will be submitted to the ethics committee, updated in the https://ensaiosclinicos.gov.br registry, and emailed to investigators. Participants will be notified if there are changes to eligibility criteria or outcomes.

### Participants' Recruitment

Participants will be recruited from the hospital's surgical waiting list or partner referrals. Eligible candidates will complete a screening form, receiving study details by phone. After agreeing, they will undergo medical evaluations and assessments for their participation in the study.

### Participants' Allocation

Participants will be allocated by a research assistant to either modified LET, ALL-S or -D, combined with ACLR. Eligibility-confirmed athletes will be assigned via computer-generated block randomization (1:1:1 ratio, blocks of six) to ensure balanced group sizes throughout enrollment. 9 Randomly ordered sequences (e.g., ABC, ACB, BAC, BCA, CAB, CBA) dictate allocation; each participant receives the next intervention in their block's sequence, maintaining equal distribution for every six enrollments.

### Eligibility Criteria

Inclusion criteria consist of: (1) being a federated athlete who competed in at least official competitions within the past 2 years; (2) between 18 and 45-years-old; (3) diagnosis of an isolated ACL injury without concomitant knee ligament damage; and (4) no prior surgery on the affected joint. The exclusion criteria were postoperative infection, inability to attend follow-up evaluations, or refusal to participate in scheduled assessments.

### Interventions

The modified LET involves harvesting a strip of the iliotibial band, which is passed deep to the lateral collateral ligament and fixed near the femoral insertion of the ALL, with the knee at 30° flexion and neutral rotation, providing rotational stability while avoiding excessive constraint. 6

The semitendinosus tendon was prepared in a triple configuration and combined with the gracilis tendon in a single-strand fashion, resulting in a four-strand graft for the intra-articular ACL reconstruction. The longer strand, derived from the gracilis, was used for ALL reconstruction.

The femoral tunnel was created using an outside-in guide, with an anatomic position for the intra-articular ACL reconstruction and an anatomic position for the extra-articular ALL reconstruction, located 5 mm posterior and proximal to the lateral epicondyle.

On the tibial side, the gracilis graft was routed distally, passing superficial to the lateral collateral ligament (LCL) and deep to the iliotibial band, and was fixed in the region between Gerdy's tubercle and the fibular head, approximately 1 cm distal to the joint line. 10

Finally, ALL-D employs the same grafts used in the LAL-U technique, with the same femoral fixation as previously described. Tibial fixation was performed through a communicating double tunnel, positioned between Gerdy's tubercle and the fibular head, approximately 1 cm distal to the joint line. This configuration resulted in a double-bundle ALL reconstruction, aiming to mimic the native fan-shaped anatomy of the ligament. 7

### Outcomes

Participant assessments will occur in two phases: preoperatively (1–2 weeks before surgery) and postoperatively during outpatient visits at 3, 6, and 9 months. Evaluations will include general medical history reviews, self-reported functionality measures, quality-of-life metrics, physical activity levels, as well as clinical measurements of joint laxity, quadriceps morphology, and knee extensor strength. Telephone follow-ups at 12 and 24 months will collect self-reported data regarding functionality, quality-of-life, and activity levels.

Four validated questionnaires will be used: the International Knee Documentation Committee's (IKDC) subjective knee form for functionality, which has demonstrated superior psychometric properties compared to alternatives; 11 the 36-Item Short Form Health Survey (SF-36) for quality-of-life, which has established normative references for the local population and proven applicability in orthopedic populations. 12 Finally, the Tegner Activity Scale was used for preinjury and current activity levels. 13 A custom-designed treatment questionnaire will track physical therapy and clinical details. Responses will be recorded electronically using de-identified forms to ensure confidentiality.

Joint laxity assessments will be performed using the KT-2000 arthrometer (MEDmetric Corp.) according to manufacturer specifications. 14 Three consecutive measurements will be obtained for each limb, with the mean values used for subsequent analysis.

Quadriceps morphology will be evaluated via standardized ultrasound imaging acquired with Mindray Z6 system (Shenzhen Mindray Bio-Medical Electronics Co. Ltd.), with a 40 mm linear transducer (7–15MHz). Transverse panoramic images will be captured from the vastus lateralis, rectus femoris, and vastus intermedius muscles. 15 Subsequent image analysis using the ImageJ (free and open source) software will quantify muscle thickness and echo intensity.

Functional performance will be assessed through the Single-Hop Test (SHT) and Y-Balance Test (YBT) to evaluate lower limb power and dynamic postural control, 16 17 following standardized protocols. For the SHT, participants stand behind a marked line, jump horizontally on one leg, and land without overstepping. The average of three trials will be analyzed. For the YBT, participants will stand barefoot on one leg at the center of a Y-shaped marking, reaching with the other leg in three directions (anterior, posteromedial, posterolateral), touching the line lightly before returning. Three trials per direction will be averaged.

Knee strength will be measured using an isokinetic dynamometer (Humac Norm II, Computer Sports Medicine Inc.) at 60°/s, with participants performing maximal effort repetitions of knee extension and flexion. 15 18 Throughout testing, the evaluator will monitor and document any reported pain, as well as peak torque values and any pain observations.

Demographic and clinical data (height, body mass, smoking status, comorbidities) will be obtained from medical records or structured interviews. All procedures ensure participant privacy while capturing relevant clinical and functional outcomes.

Follow-up reminders via phone/email and flexible scheduling will be used to promote participant retention. Outcome data (IKDC, SF-36, Tegner scales, and reinjury rates) will be collected via telephone for participants who discontinue the physical assessment but consent to follow-up.

### Sample Size

The sample size was calculated for a mixed model analysis comparing three groups (LET, ALL-S, and ALL-D) across six time-points (preintervention and 3-, 6-, 9-, 12-, and 24-months postintervention), adjusting for meniscal repair status. Based on an analysis of covariance (ANCOVA) model with an effect size of f = 0.25, alpha = 5%, and 80% power (G*Power 3.1.9.2, Heinrich-Heine-Universität Düsseldorf), the estimate was N = 220. Missing data will be handled using imputation methods.

### Blinding

This single-blind trial ensures blinding only for the researcher handling data collection and analysis, while surgeons, participants, and caregivers remain aware of treatment allocations to maintain objectivity.

### Statistical Analysis

Statistical analysis will use linear mixed-effects models to assess intervention group, time, and their interaction effects, adjusting for meniscal repair status as a covariate. The model includes fixed factors for group (0 = LET, 1 = ALL-S, 2 = ALL-D), timepoint (baseline, 3, 6, 9, 12, and 24-months), and meniscal status (0 = absent, 1 = present), along with their interactions, plus random participant intercepts. Model assumptions (normality, homoscedasticity) will be checked via Q-Q plots and Levene's tests.

The post hoc comparisons of marginal means will apply Bonferroni correction across groups and timepoints. All analysis will be implemented in Python (Python Software Foundation) environment using the *statsmodels* package.

### Data Management and Monitoring

Double data entry will be performed by independent researchers to minimize errors, followed by continuous checks for outliers. Electronic forms will be stored in restricted databases, accessible to the PI, statisticians, and regulatory authorities. The research staff will review safety and adherence concerns annually, monitoring adverse events and protocol deviations. Interim analyses are planned for 50% enrollment (N = 110). Any adverse events will be recorded and reported to the ethics committee using standardized forms.

## Discussion

This study protocol presents a prospective clinical trial designed to evaluate and compare two surgical techniques for ACLR combined with extra-articular reinforcement in high-performance athletes. The investigation of modified LET, 6 and ALL-S and ALL-D, 10 will provide important insights into optimizing functional recovery and return to sport following ACL injury.

The expected findings from this trial may significantly advance current understanding of how different surgical approaches influence postoperative outcomes in athletic populations. To ensure broad dissemination, results will be published open-access and shared via social media. The full protocol, statistical code, and participant-level data will be made available upon reasonable request.

The clinical relevance of this study lies in its potential to clarify which extra-articular reinforcement technique may be most suitable for different athlete profiles. Based on existing biomechanical evidence, we hypothesize that LET may demonstrate advantages in early-stage dynamic stability due to its immediate restoration of mechanical stability in knee anterior translation and internal rotation. 19 In contrast, all reconstruction techniques (ALL-S and ALL-D) are hypothesized to yield superior long-term outcomes in strength restoration and functional performance by more accurately replicating native knee biomechanics. 7 These anticipated findings could help clinicians make better informed decisions when selecting surgical techniques based on an athlete's specific sport demands and rehabilitation goals.

This protocol builds upon and extends current literature in several important ways. While previous studies have established the benefits of extra-articular procedures in reducing rotational laxity and graft failure rates compared to isolated ACLR, there remains limited high-quality evidence directly comparing different reinforcement techniques in athletic populations. 20 The inclusion of comprehensive functional assessments at multiple time points will allow for a detailed examination of recovery trajectories, potentially identifying critical windows for targeted rehabilitation interventions. Additionally, by systematically accounting for meniscal status as a covariate, this study is designed to help resolve ongoing debates about how concomitant meniscal repair influences postoperative recovery timelines.

The methodological approach incorporates several features designed to ensure robust and reliable findings. The longitudinal design with assessments scheduled for several time-points postoperatively will capture both short-term functional recovery and longer-term outcomes relevant to athletic performance. The combination of patient-reported outcome measures with objective physical performance tests provides a multidimensional evaluation of recovery that reflects both clinical and functional perspectives.

Standardized surgical protocols and rehabilitation guidelines have been implemented to minimize variability in technique execution and postoperative care across treatment groups. While complete blinding of participants and surgeons is not feasible due to the nature of the interventions, the use of blinded outcome assessors and statisticians will help maintain objectivity in data collection and analysis.

Several potential limitations warrant consideration in the interpretation of future results. The quasirandomized design, while practical for surgical trials, may introduce selection bias that could influence outcomes. To address this, detailed baseline characteristics will be collected and accounted for in the statistical analysis. The focus on high-performance athletes, while appropriate for studying high-demand individuals, may limit generalizability to recreational athletes or nonathletic populations. The extended follow-up period necessary to assess long-term outcomes may lead to participant attrition, though strategies such as telephone follow-ups and incentives have been incorporated to enhance retention.

## Conclusion

This protocol describes a comprehensive clinical trial that addresses important gaps in current knowledge regarding optimal surgical approaches for ACLR in athletic populations. By systematically comparing LET, ALL-S, and ALL-D techniques, the study aims to provide evidence that can guide clinical decision-making and potentially establish more standardized approaches to managing ACL injuries in high-performance athletes. Upon completion, the findings may influence surgical technique selection, rehabilitation protocols, and ultimately improve outcomes for athletes returning to demanding sports activities.
